# The Social Context of Women's Health

**DOI:** 10.1186/1472-6874-4-S1-S2

**Published:** 2004-08-25

**Authors:** Vivienne Walters

**Affiliations:** 1 National Centre for Public Policy, University of Wales Swansea, Swansea, UK

## Abstract

**Health Issue:**

The discussion of health emphasizes the importance of analyses of social determinants of health. Social determinants permit the targeting of policies towards the social factors that impair or improve health. Two broad questions are considered: (i) what do we know about the social determinants of women's health? (ii) are there gender-related differences in health problems, and how we might explain them?

**Key Findings:**

While 'sex' may be used to denote the biological difference between women and men, it is an imperfect measure of 'gender'. It is argued that a single measure cannot hope to capture the complexity of gender nor the ways in which gender relations change over time and give rise to or exacerbate health problems. The literature on the social determinants of health shows the importance of placing a primary emphasis on addressing the social and economic sources of ill health at national, provincial and community levels.

**Data Gaps and Recommendations:**

Recent studies of gender differences in health point to a lack of data and to the importance of understanding changing gender relations; differences in power and access to resources between women and men, and changing expectations of appropriate gender roles and behaviours. Poverty, social exclusion, unemployment, poor working conditions and unequal gender relations have a profound influence on patterns of health and illness. We suggest some material markers of change that might be used in health surveillance. With a more complete understanding of gender's role in shaping daily lives, these markers could be refined and expanded.

## Introduction

Data analyses are meaningful when guided by conceptual frameworks. This chapter sets a context for this report by highlighting the importance of gender and the links between gender and health. The focus is on the social determinants of health. The ways in which we understand the relation between gender and health have implications for strategies of change and for policy making, and they provide a guide for future research, data collection and health surveillance by pointing to gaps in existing data.

The chapter starts by considering some key dimensions of gender differences and the inequalities that characterize gender relations. These indicate that while "sex" may be used to denote the biological difference between women and men, it is an imperfect measure of gender. A discussion of health follows, with a focus on the importance of analyses of the social determinants of health. This discussion then leads into a consideration of two broad questions: (i) What do we know about the social determinants of women's and men's health? and (ii) Are there differences in the health problems women and men experience and, if so, how might we explain them? Recent studies help to provide partial answers, and they also point to the types of research needed in the future as well as some of the measures that might serve both as indicators of changing gender relations in Canada and as a basis for health surveillance. In conclusion, the policy implications of this discussion are emphasized and directions for future research proposed.

### The Importance of Gender

Gender matters. [1] Being born a boy or a girl has a profound influence on the shape of an infant's future life. Compared with men, women are less likely to be employed full time, more likely to be attuned to caring roles, and more likely to have their working life interrupted by pregnancy and caring responsibilities. Women generally work in lower-paid jobs, and they exercise less control in those jobs. Research also tells us that women's views are more likely to be devalued, women are less likely to occupy top positions in society, and women are more likely to be seen as irrational, emotional and unsuited for responsible positions. Even though women have entered the labour force in greater numbers, they still assume most of the responsibility for household chores. Women's economic dependence on men is signified by the dramatic change in their lives after divorce or separation. It is not surprising that women also have lower self-esteem and are more likely to be concerned about body image (see the chapter entitled "Body Weight and Body Image").

Women's lives are also shaped by race and ethnicity, by their sexual orientation, and by age and their stage in the life cycle. These represent diverse structures of inequality that, along with gender, can compound the disadvantage and discrimination that women still face. Among the most vulnerable are older women and lone parents (both of whom are most likely to be living below or close to the poverty line) as well as women in low-paid jobs and those who suffer racial discrimination. All women are at risk of violence, especially at the hands of their male partners, and this is one of the more extreme manifestations of the power imbalance that marks gender relations.

In many respects the features of women's day-to-day lives have been almost invisible and often taken for granted. The women's movement and feminist research have been important in helping to highlight what women do and the ways in which gender relations help to perpetuate the disadvantage women experience. One key contribution is the recognition of household responsibilities as domestic labour or unpaid work that makes heavy demands on women's time and represents an important economic contribution. [2] It is more than a labour of love. Yet we still have not developed ways of identifying the components and character of this work. [3,4] While time budget surveys are moving in this direction, it is still rare for government surveys to measure the content, burden or contribution of this work. Similarly, the vast body of research that has focused on the social organization of paid work has seldom paid attention to the conceptualization of domestic labour.

Another important body of literature is that which documents the nature of women's paid work roles. From much earlier research that asked whether women's employment had an adverse effect on the health of children and the welfare of the family (a response to women's entering the labour force in greater numbers), we have moved to looking at women as workers, at the social organization of the work that they do, and at the physical and psychosocial hazards that they face in the workplace. [5,6] In turn, this new focus has opened up the possibility of comparing the impact of work roles on both women and men, though this continues to be methodologically challenging, partly because of gender segregation in the labour market. [7] A more recent strand in this literature is that which looks at the effects of restructuring. [8,9] Because many women work in the public sector, they are affected by cutbacks and organizational changes in the health and education sectors and in municipal government; moreover, increased workloads and cutbacks in services affect women both as workers in, and consumers of, public services.

This report presents information from national data sources on gender and health. As the following chapters will reveal, the existing data are often incomplete, unavailable or not presented in the most meaningful form. In most instances, official statistics include a breakdown by sex but do not provide sufficient measures to allow exploration of the influence of gender. As this section suggests, gender refers to a complex web of changing roles and relations, and the use of sex as a proxy for gender will yield a very limited understanding of the way gender and health are linked. We need to develop other measures that are based on a much finer appreciation of how women's and men's lives are structured by gender, and these in turn can be reflected in women's health surveillance and other policy initiatives.

### Population Health: The Social Determinants of Health

It has long been recognized that the conditions in which people work and live affect their health. [10] This has informed the growth of public health, and at one point in the nineteenth century such ideas challenged an emergent biomedicine. [11] For well over a century there has been strong documentation of the links between mortality and economic development, income and living standards. Public health initiatives led to significant improvements in infant mortality and life expectancy before biomedicine had an impact on the health of populations. [12] In more recent decades the same links have been established with respect to morbidity. For example, Marmot and Wilkinson and their colleagues have provided extensive documentation of the health effects through the life course of poverty, social exclusion and minority status, unemployment and job insecurity, the social organization of work, social support and social cohesion, transport, food and tobacco. [13] Some of the most recent research has emphasized the importance of the degree of income inequality rather than levels of income *per se*. [14] Yet, despite this emphasis on the social aspects of health, there has been remarkably little attention to the role of gender in this literature. [15,16] Macintyre [17] illustrates the ways in which gender has often been neglected in studies and shows how this has been to the detriment of both women and men. If we were to include gender in analyses, she argues, we would also have a better understanding of socio-economic differences in health. The socio-economic gradient in various measures of health is less marked for women (with the exception of coronary heart disease and body shape) and, rather than being an artifact, this may signify gender differences in exposure or vulnerability. One recent analysis of British data stands out because it focuses on both gender and ethnicity in relation to socio-economic status and health. It reveals considerable differences in health status between men and women within majority and minority ethnic groups. [18]

The literature on the social determinants of health has been important in drawing attention to the social causes of ill health. In drawing out the implications of this model, McKinlay [19] has argued that it is important to focus "upstream" on what is causing illness in the first place, rather than simply treating the sick bodies pulled out of the stream. Such a focus opens up the possibility of more meaningful surveillance of the social factors that contribute to poor health among women and among men, so that "upstream" interventions can be better monitored and targeted.

These observations lead into a discussion of broad themes in research on gender and health – the social determinants of women's and men's health and the extent to which there are differences in the health problems women and men experience.

### Linking Gender and Health

1. What do we know about the social determinants of women's and men's health? To what extent can their health be explained by behavioural influences and to what extent are social structural influences crucial?

These questions are important because they can help to shape strategies for change and health surveillance activities. The British Health and Lifestyles Survey in the 1980s weighed evidence on key determinants of health and argued that women's and men's circumstances appeared to have a more profound effect on their health than their behaviours did. [20] Analysis of data from the National Population Health Survey (NPHS) in the chapters of this report confirms that social structural factors such as income, education, occupation, family structure and social support are especially important in explaining women's and men's health (see, for example, the chapter on "Multiple Roles and Women's Mental Health in Canada"). These factors shape health directly and also influence individual behaviours such as smoking, drinking, weight and physical activity. [21,22] Other chapters describing the ways in which behaviours are influenced by the broader social context point out that women in vulnerable positions, such as single parents and Aboriginal women, are at particular risk of personal and chronic stress, and poor health outcomes (see the chapters entitled "Multiple Roles and Women's Mental Health in Canada," "Depression," "Violence against Canadian Women").

Graham argues that smoking is concentrated among those in the most disadvantaged positions. [23,24] Her research has shown that, among women, tobacco consumption is highest for those facing high levels of stress – young mothers, particularly those who are lone parents. Among men, alcohol appears to be a common way of coping with disadvantage and stress. Women and men turn to different coping mechanisms, all of which can impair their health while making no impression on the social and material conditions that give rise to these behaviours. Such "unhealthy" lifestyles are culturally appropriate responses to the social context that prompts depression and despair.

If we wish to prevent health problems, our most fundamental focus must be on the social and material conditions of men's and women's lives. McKeown [12] and, most recently, Townsend [25] in his blueprint for reform of health services in Wales, have argued that the biggest impact on inequalities in health will come from addressing the underlying socio-economic determinants of poor health. This means that it is important to develop indices of social support and social cohesion as well as to monitor key issues such as:

• unemployment

• poverty

• benefit levels

• housing conditions

• food security

• working conditions

• regional disparities

• populations that may be at particular risk of material deprivation and social exclusion (women, the elderly, indigenous peoples, racial and ethnic minorities, lone-parent families).

Evidence in many countries spanning more than a century suggests that such material factors are crucial in understanding levels of population health and the reasons some women and men are at greater risk of disease, disability and death. Yet this is not a call for reduced investment in the health care sector. Even though the bulk of preventive strategies lie outside the health sector, health care can alleviate symptoms and influence the severity of disease. [25] As McKeown [26] has argued:

The conclusion that medical intervention is often less effective than has been thought in no way diminishes the clinical function. When people are ill they want all that is possible to be done for them and small benefits are welcome when larger ones are not available.

It is crucial that those most at risk of ill health are identified and encouraged to make use of whatever screening, preventive, diagnostic and treatment services have been shown to be effective. Moreover, insofar as health services can establish alliances with other sectors to tackle the social bases of illness, all of these sectors may be in a better position to influence levels of health. Structural links at the most senior levels of health and other sectors can provide the impetus for joint strategies and encourage collaboration at the community level among those addressing issues such as health, poverty, housing and community development.

Policies and services that address health promotion at the individual level by seeking to achieve behavioural change are also important. Efforts to improve health should focus on both the individual and the societal levels. The latter is emphasized here because the social structural determinants of health have so often been neglected in debates about health. The challenge we face is to develop frameworks of understanding that allow us to see pathways of influence from the societal level to individuals and their experience of illness, and to document how the influence flows in both directions – the ways in which the biological experience of disease has social costs.

2. Are there differences in the health problems women and men experience and, if so, how might we explain them?

The common belief has been that "women are sicker but men die quicker": that women are more likely to report health problems whereas men have a shorter life expectancy. Other recent research shows that gender differences in health are less clear than is often assumed. [4,27,28] The general measures of health status as well as the specific measures of mental and physical health problems used in the NPHS indicate different patterns with respect to gender: in some cases no differences between women and men; in others, small or inconsistent differences. Yet women are more likely to report short-term disability, distress, depression, migraine, pain, arthritis or rheumatism, and non-food allergies. [4]

Such observations in several countries have led to calls for much more attention to gender and the changing nature of gender roles. It has been argued that with changes in gender roles and the recognition of diversity among both men and women, some men may have more in common with some women. [28] But there *are *some fairly consistent gender differences, and we need greater documentation of these as well as of the ways in which men's health and women's health are similar. To understand such data and to lay the basis for meaningful analysis of gender and health, it is important to chart gender relations over time.

We do not know enough about how gender relations have been changing over the past few decades, and this complicates the task of tracing links between health and gender. [1] Women have entered the labour market in greater numbers, though they are typically employed in part-time work and in lower-paid "women's jobs," which often allow workers less autonomy and control in their work. While women may now have a greater degree of economic independence than previously, their relation with the labour market is still weaker than that of men. However, men, who at one time could expect almost continuous employment until retirement at 65, now face the prospect of redundancies and long-term unemployment as a result of restructuring and changes in the labour market. Charles has argued that these and other changes in gender relations mean that the "old ways of being a man are no longer possible." [29]. The increase in divorce rates has had a profound effect on many women, who are immediately upon divorce faced with a considerable drop in household income and in their command of other resources. As lone parents they are at high risk of living in poverty.

For women and men who have jobs, there is evidence that, as a result of restructuring, workloads have intensified and this may have critical effects on health. Women working in the public sector are doubly affected. As workers they face a heavier workload and heightened job insecurity and, with cuts in the provision of public services, there are fewer services to support them in their domestic and caring roles. Restructuring in the name of efficiency has created a care deficit in the home. A recent report on the progress of the world's women has argued for more holistic definitions of "efficiency" that take into account more than financial costs and recognize the value of women's work. [30] Provision of affordable child care is still woefully inadequate, and this also has implications for women, who continue to be defined as the main caregivers.

These changes in job security, family structure, income levels, dependence on benefits and availability of public services are all key aspects of gender relations. They shape expectations of men's and women's roles and the resources that are available to them to meet these expectations. When sex is used in the analysis of health data it serves as a proxy for gender, and these are some of the unwritten, unspecified elements of gender relations. The problem we face is that such a single measure cannot hope to capture the complexity of gender or the ways in which gender relations change over time and give rise to or exacerbate health problems. [31]

One step in charting changing gender relations is to document broad changes in the position of women in Canadian society. A starting point for this is the approach adopted by the United Nations in a global monitoring of the status of women. [30] This shows Canada in the context of other countries at various stages of economic development. The indicators used include the following:

• female enrolment in secondary education

• women's participation in the labour force by sector

• female share of administrative and managerial positions

• female share of seats in national parliament

• female wages as a percentage of male wages by sector

• gender-poverty ratio

• prevalence of violence against women

• prevalence of HIV/AIDS

To these could be added others that document the availability of child care and the gender division of tasks within the home. Yet these indices cannot provide a full understanding of the nature of gender relations, and so researchers have called for more qualitative research, which can better capture the nuances of gender and which might lead to the development of better indices. [4,7,28,32]

Focusing on gender in this way requires a redefinition of our approach to understanding health. To this point, the dominant emphasis has been on biomedical interventions in relation to the individual, and these have been guided by a disease-based model. But if the targets for intervention are socio-economic, then we have to take features of social life as a starting point. We must place a primary emphasis on fighting poverty, social exclusion, unemployment, poor working conditions and gender inequalities, each of which can influence lifestyles and prompt or exacerbate a range of different health problems. Doyal [15] points the way to such an emphasis

traditional epidemiological methods have to be turned on their head. Instead of identifying diseases and then searching for a cause, we need to begin by identifying the major areas of activity that constitute women's lives. We can then go on to analyse the impact of these activities on their health and well-being.

This is not to dismiss the role of medicine and biological sciences, for we need to understand how the social is embodied, and we must respond to chronic and acute diseases. It does, however, call for a major change in the ways we think about health, and it opens up the opportunity for communities to define problems and generate responses to them.

### Rethinking our Approach to Health and Illness: Implications for Research and Policy

Above all, it is important that we place a primary emphasis on addressing the social and economic sources of ill health at national, provincial and community levels, as these will prevent more deaths and chronic illness than any health care interventions. Poverty, social exclusion, unemployment, poor working conditions and gender inequalities have a profound influence on patterns of health and illness. Health care policy is important, but it is only one element of the necessary public policy response, yet research attentive to the social structuring of women's health can contribute knowledge relevant to this wider array of policy domains. Policy-making will require much greater collaboration between government departments and others concerned with social exclusion and inequality.

At the same time, we need to develop our understanding of changing gender relations, differences in power and access to resources between women and men, and changing expectations of appropriate gender roles and behaviours. This chapter has suggested some material markers of change, although with a fuller understanding of how gender shapes people's day-to-day lives these measures could be refined and expanded. In so doing, it is best to use both quantitative and qualitative research, as each type of data will capture different aspects of gender.

In developing strategies to reduce the most obvious and unacceptable inequalities, liaison with communities is key. Insofar as problems of poverty, social exclusion and gender inequalities can be addressed at the local level, this should be in concert with local groups. If health is, in large part, created by the social environment, then that social context must be the first point of intervention in preventing problems. Health services can play an important role by identifying those most at risk in communities and ensuring that they receive the screening, diagnostic services or treatments that are effective.

In tracing the ways in which women's and men's life experiences are "written" on their bodies – the way the social is embodied – social and biological sciences must work alongside each other, tracing the ways in which women's and men's lives help to create or exacerbate health problems. This would feed back into policies regarding gender and socio-economic inequalities and would also inform other curative or coping responses.

This report draws attention to the importance of women's health surveillance and points to some of the ways in which women's health and women's lives can be documented with the aim of improving interventions. The following chapters document the general health status of women and serves as a prelude to the discussion of health determinants and health care utilization.
